# Supplementary material for: The giant pouched rat (Cricetomys ansorgei) olfactory receptor repertoire
Source: PLoS One. 2020 Apr 2;15(4):e0221981. doi: 10.1371/journal.pone.0221981 (PMC7117715; doi:10.1371/journal.pone.0221981)
Supplement: S1 File — (DOCX) [file pone.0221981.s001.docx]

**ORA Method**

This method uses hidden Markov models (HMMER) (1) to identify and categorize ORs into 13 gene families, and categorizes OR genes as functional when they are at least 650bp in length with no stop codon (2). As input, we included OR gene sequences collected as above from Ensembl BioMart for rat and mouse, and obtained OR gene sequences for the squirrel from Hughes (2018, Graham Hughes, pers. comm). We then processed the resultant ORA output by removing duplicate sequences. The ORA module identified 3585 unique pouched rat OR genes total, 2525 of those were identified as pseudogenes. The discrepancy in these two measures (i.e. manual pipeline versus ORA) is largely due to an increased identification of short truncated sequences using the ORA method; only 31 of these pseudogenes were greater than 750 bp. Using identical filtering in processing ORA data, as was used in processing the manual pipeline search method, would have identified 20 of these 2525 ‘pseudogenes’ as intact genes due to length, whereas only 30 of these pseudogenes would have been identified as non-spurious pseudogenes. Thus, the module detected 1060 unique putative functional OR receptor genes from the pouched rat genome.

**Result**

This method identified 1060 functional OR genes, and 2525 pseudogenes, with a large proportion of these due to short truncates. The ORA method organized all pouched rat ORs into 16 families (S1 Table), with the 5/8/9 family containing the largest number of functional and pseudogene ORs.

*Orthology*

Generally, most families or family groups (as defined by the ORA method) had a small percentage of orthologs, and the overall contributions of orthologs were similar (**Supplementary Figure 1**). However, some smaller families (OR families 12, 14, 55, and 56) had no 1-to-1 orthologs with the other rodents in this study. Families 51 and 52 tended to have larger percentages of orthologous genes (31-37%), and OR family 11 had all three representative genes share orthologs with the other rodents (**Supplementary Figure 1**).

1. Finn RD, Clements J, Eddy SR. HMMER web server: interactive sequence similarity searching. Nucleic Acids Res. 2011 Jul;39(Web Server issue):W29-37.

2. Hayden S, Bekaert M, Crider TA, Mariani S, Murphy WJ, Teeling EC. Ecological adaptation determines functional mammalian olfactory subgenomes. Genome Res. 2010 Jan 1;20(1):1–9.
