# Supplementary material for: The giant pouched rat (Cricetomys ansorgei) olfactory receptor repertoire
Source: PLoS One. 2020 Apr 2;15(4):e0221981. doi: 10.1371/journal.pone.0221981 (PMC7117715; doi:10.1371/journal.pone.0221981)
Supplement: S1 Fig — (DOCX) [file pone.0221981.s008.docx]

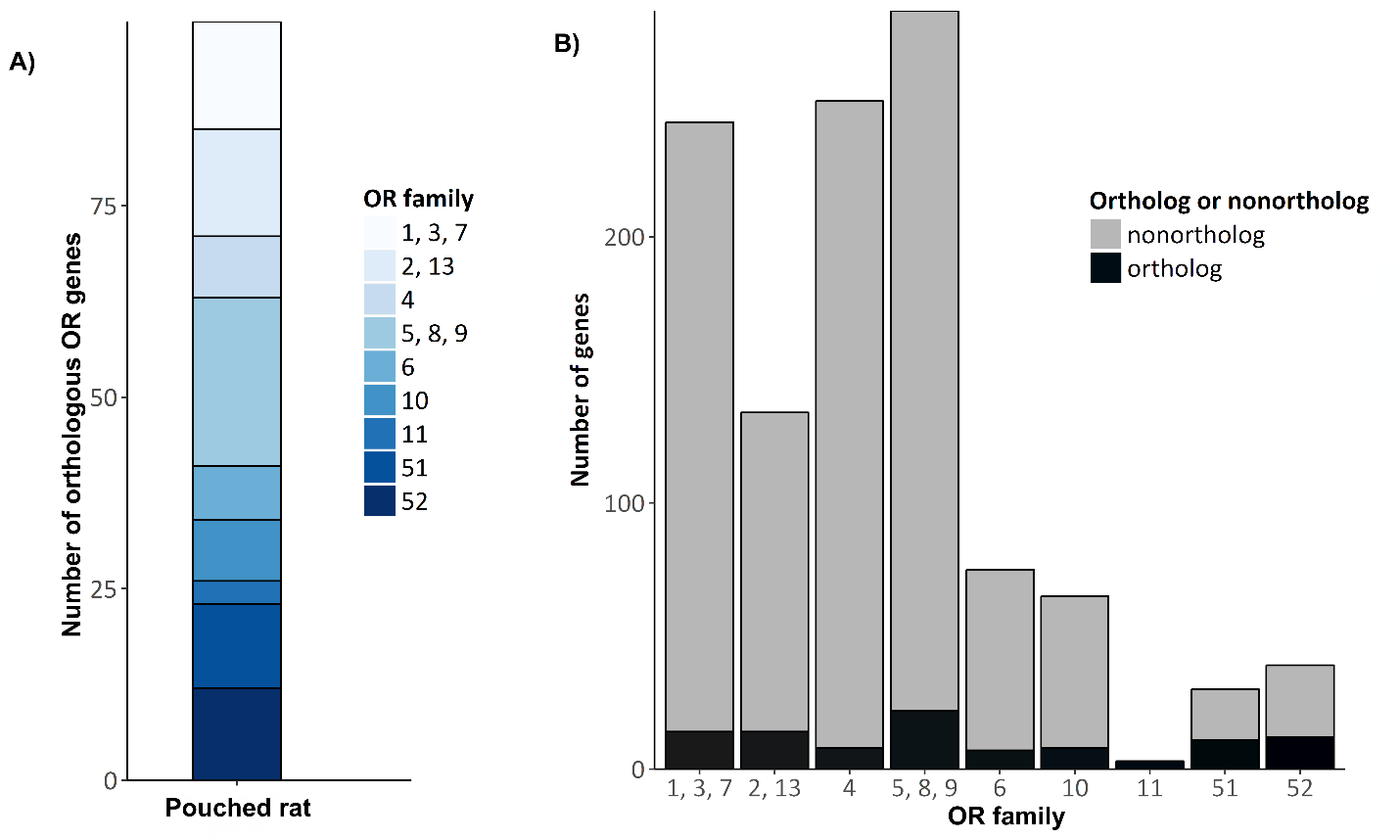


**S1 Figure Orthologous pouched rat olfactory receptor (OR) genes by family grouping (as defined by olfactory receptor assigner (ORA) method).** A) Representation of pouched rat 1-to-1 orthologs to mouse, rat, and squirrel across family groups B) Stacked bar chart of 1-to-1 orthologs and all other pouched rat OR genes within each family. ORA families 12, 14, 55 and 56 are not shown since they contained no orthologous genes.
